# Microglia are effector cells of CD47-SIRPα antiphagocytic axis disruption against glioblastoma

**DOI:** 10.1073/pnas.1721434116

**Published:** 2019-01-02

**Authors:** Gregor Hutter, Johanna Theruvath, Claus Moritz Graef, Michael Zhang, Matthew Kenneth Schoen, Eva Maria Manz, Mariko L. Bennett, Andrew Olson, Tej D. Azad, Rahul Sinha, Carmel Chan, Suzana Assad Kahn, Sharareh Gholamin, Christy Wilson, Gerald Grant, Joy He, Irving L. Weissman, Siddhartha S. Mitra, Samuel H. Cheshier

**Affiliations:** ^a^Division of Pediatric Neurosurgery, Department of Neurosurgery, Lucile Packard Children’s Hospital, Stanford University School of Medicine, Stanford, CA 94305;; ^b^Institute for Stem Cell Biology and Regenerative Medicine, Stanford University School of Medicine, Stanford, CA 94305;; ^c^Ludwig Center for Cancer Stem Cell Research and Medicine at Stanford, Stanford University School of Medicine, Stanford, CA 94305;; ^d^Department of Neurosurgery, University Hospital Basel, CH-4031 Basel, Switzerland;; ^e^Department of Neurobiology, Stanford University School of Medicine, Stanford, CA 94305;; ^f^Neuroscience Microscopy Center, Wu Tsai Neurosciences Institute, Stanford University, Stanford, CA 94305;; ^g^Department of Radiology, Stanford University School of Medicine, Stanford, CA 94305;; ^h^Department of Pediatrics, Morgan Adams Foundation Pediatric Brain Tumor Research Program, Children’s Hospital Colorado, University of Colorado Anschutz Medical Campus, Aurora, CO 80045;; ^i^Division of Pediatric Neurosurgery, Department of Neurosurgery, Huntsman Cancer Institute, University of Utah, Salt Lake City, UT 84112

**Keywords:** anti-CD47, microglia, glioblastoma, immunotherapy, checkpoint inhibition

## Abstract

Glioblastoma multiforme (GBM) is a highly aggressive malignant brain tumor with fatal outcome. Tumor-associated macrophages and microglia (TAMs) have been found to be major tumor-promoting immune cells in the tumor microenvironment. Hence, modulation and reeducation of tumor-associated macrophages and microglia in GBM is considered a promising antitumor strategy. Resident microglia and invading macrophages have been shown to have distinct origin and function. Whereas yolk sac-derived microglia reside in the brain, blood-derived monocytes invade the central nervous system only under pathological conditions like tumor formation. We recently showed that disruption of the SIRPα-CD47 signaling axis is efficacious against various brain tumors including GBM primarily by inducing tumor phagocytosis. However, most effects are attributed to macrophages recruited from the periphery but the role of the brain resident microglia is unknown. Here, we sought to utilize a model to distinguish resident microglia and peripheral macrophages within the GBM-TAM pool, using orthotopically xenografted, immunodeficient, and syngeneic mouse models with genetically color-coded macrophages (*Ccr2*^RFP^) and microglia (*Cx3cr1*^GFP^). We show that even in the absence of phagocytizing macrophages (*Ccr2*^RFP/RFP^), microglia are effector cells of tumor cell phagocytosis in response to anti-CD47 blockade. Additionally, macrophages and microglia show distinct morphological and transcriptional changes. Importantly, the transcriptional profile of microglia shows less of an inflammatory response which makes them a promising target for clinical applications.

## Significance

Tumor-associated microglia and macrophages (TAMs) constitute up to one half of the cells in glioblastoma multiforme (GBM) and are known to promote tumor growth. Therefore, modulation and reeducation of the TAM pool is a promising antitumor strategy against GBMs. We recently showed that disruption of the SIRPα-CD47 signaling axis is efficacious against various brain tumors including GBM primarily by inducing tumor phagocytosis. Here, we show that tumor-associated microglia are capable of in vivo tumor cell phagocytosis in response to anti-CD47 blockade. The activation of microglia was associated with distinct morphological and transcriptional changes. In fact, microglia show a dampened inflammatory response upon anti-CD47 therapy compared with macrophages, making them an attractive target for clinical applications especially in the confined regions of the brain.

Glioblastoma multiforme (GBM, World Health Organization grade IV) is a fatal brain tumor resistant to all conventional treatment (1). A significant proportion of the tumor mass is made up of nonneoplastic cells which play a major role in tumor growth and progression. Recent studies show complex interactions between tumor cells and the microenvironment, highlighting the crucial role of nonneoplastic cells for therapy response (2). The pool of tumor-associated macrophages (TAMs) accounts for the majority of nonneoplastic cells including peripheral macrophages from hematopoietic stem cells (HSCs) and brain-resident microglia which originate from immature yolk sac progenitors (3, 4). In the tumor environment TAMs have immune suppressive functions. The fact that as many as 30–50% of the cells in gliomas are microglia and macrophages raises the intriguing potential of reeducating tumor-associated microglia (TA-MG) and macrophages (TA-MAC) to act as antitumor effector cells (5). Numerous studies have demonstrated the distinct roles of microglia and macrophages in CNS diseases (6, 7). A well-characterized mechanism of immune evasion by tumor cells is the up-regulation of the antiphagocytic (“don’t eat me”) surface protein CD47 which binds to its cognate receptor SIRPα on phagocytic cells inhibiting its phagocytic activity (8). CD47-SIRPα myeloid checkpoint blockade has been shown to effectively enhance tumor phagocytosis and hence reduce tumor burden (9, 10). However, most effects are attributed to macrophages recruited from the periphery, but the role of the brain resident microglia is unknown (10). Genetic lineage tracing experiments revealed that expression of the chemokine receptor CCR2 is restricted to inflammatory monocytes and macrophages, whereas the fractalkine receptor CX3CR1 is highly expressed by microglia from early development (11). In consequence, *Ccr2*^*RFP/wt*^*-Cx3cr1*^*GFP/wt*^ reporter mice have been used to differentiate between macrophages and microglia (6). Here, we created a genetically color-coded mouse xenograft model (*Ccr2*^*RFP/wt*^*-Cx3cr1*^*GFP/wt*^) on a NOD.Cg-Prkdc^*scid*^:Il2rgtm1^*Wjl/SzJ*^ background, which allowed us to decipher the differential contribution of microglia and macrophages to the GBM-TAM pool at baseline and their response to the myeloid checkpoint inhibitor, anti-CD47. We found microglia capable of tumor cell phagocytosis, even in the absence of phagocytizing macrophages. Additionally, microglia show distinct morphological and transcriptional changes with a less inflammatory response. CD47 blockade can effectively reeducate microglia in the GBM tumor microenvironment to unleash the therapeutic potential of tumor cell phagocytosis.

## Results

### NSG-*Ccr2*^*RFP/wt*^*-Cx3cr1*^*GFP/wt*^ Mice Were Generated and Validated to Distinguish Between Macrophages and Microglia in a Human GBM Xenograft Model.

To distinguish TA-MG from TA-MAC within the tumor environment, we created a genetically color-coded mouse xenograft model (*Ccr2*^*RFP/wt*^*-Cx3cr1*^*GFP/wt*^) on a NOD.Cg-Prkdc^*scid*^:Il2rgtm1^*Wjl/SzJ*^ background (*SI Appendix*, Fig. S1 *A*–*C*). These mice lack B, T, and natural killer (NK) cells but retain functioning macrophages and microglia. To determine if our NSG-*Ccr2*^*RFP/wt*^*-Cx3cr1*^*GFP/wt*^ mouse model allows for a robust distinction between TA-MG and TA-MAC, we validated our model by RNA-sequencing as previous reports suggest a tumor-dependent transcriptional regulation of microglia- and macrophage-specific markers (12). NSG-*Ccr2*^*RFP/wt*^*-Cx3cr1*^*GFP/wt*^ mice were orthotopically engrafted with T387 glioma cells expressing EBFP2-luciferase, and tumor engraftment was confirmed by bioluminescence imaging. After 25 d of tumor growth, TA-MG (defined as GFP^high^RFP^negative^) and TA-MAC (defined as GFP^low^RFP^high^) were sorted from dissociated xenografts by flow cytometry and processed for transcriptome analysis by RNA-seq (gating scheme: *SI Appendix*, Fig. S3). Principal component analysis and the heatmap of sample-to-sample distances showed distinct clusters for microglia and macrophages (*SI Appendix*, Fig. S2 *A* and *B*). Specifically, TA-MG were enriched for microglia-specific genes such as *Tmem119*, *Sall1*, and Pros1 compared with TA-MAC (*SI Appendix*, Fig. S2*C*). TA-MAC, on the other hand, expressed significantly higher levels of *Itga4* and *Itga1* which have recently been reported to be robust markers for glioma-associated macrophages (*SI Appendix*, Fig. S2*C*) (12). Ccr2 loss has been shown to prevent CNS infiltration by macrophages (13). Hence, we used *Ccr2* knockout mice (NSG-*Ccr2*^*RFP/RFP*^*-Cx3cr1*^*GFP/wt*^ mice) to investigate the role of TA-MG in absence of TA-MAC.

### The Microglial Composition of T387 Human GBM Xenografts Does Not Change in Response to Anti-CD47.

**Fig. 1.**
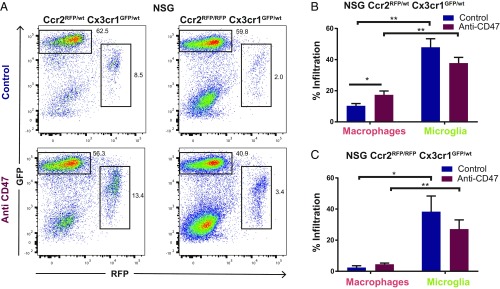
TAM composition of T387 grafted NSG-*Ccr2*^*RFP/wt*^*Cx3cr1*^*GFP/wt*^ and *Ccr2*^*RFP/RFP*^*Cx3cr1*^*GFP/wt*^ mice is dominated by microglia. (*A*) Representative FACS plots of the TAM pool in T387-EBFP2-luc grafted NSG-*Ccr2*^*RFP/wt*^*Cx3cr1*^*GFP/wt*^ and NSG-*Ccr2*^*RFP/RFP*^*Cx3cr1*^*GFP/wt*^ mice, gated on CD45 positive cells. (*B* and *C*) Composition of the TAM pool in T387-EBFP2-luc grafted (*B*) NSG-*Ccr2*^*RFP/wt*^*Cx3cr1*^*GFP/wt*^ (*P = 0.018 and **P < 0.0001) and (*C*) NSG-*Ccr2*^*RFP/RFP*^*Cx3cr1*^*GFP/wt*^ mice as assessed by flow cytometry analyses on RFP^negative^GFP^bright^ (microglia) and GFP^low^RFP^+^ (macrophages) signal gated on CD45 positive cells. *P = 0.036; **P = 0.030. Results are pooled from three independent experiments (NSG-*Ccr2*^*RFP/wt*^*Cx3cr1*^*GFP/wt*^ control group *n* = 11, anti-CD47 group *n* = 15) (NSG-*Ccr2*^*RFP/RFP*^*Cx3cr1*^*GFP/wt*^ control group *n* = 11, anti-CD47 group *n* = 10). Mean ± SEM.

Utilizing this model, we investigated the tumor microenvironment and its response to myeloid checkpoint inhibition by using the humanized anti-CD47 monoclonal antibody Hu5F9-G4 (14). NSG-*Ccr2*^*RFP/wt*^*-Cx3cr1*^*GFP/wt*^ mice were orthotopically engrafted with T387 glioma cells expressing EBFP2-luciferase. After confirming tumor engraftment by bioluminescence imaging, we started treatment with anti-CD47 (250 µg Hu5F9-G4 three times a week) or human IgG control and analyzed the tumor environment after 25 d by flow cytometry. The GBM-TAM composition in NSG-*Ccr2*^*RFP/wt*^*-Cx3cr1*^*GFP/wt*^ mice was predominantly populated by microglia (GFP^high^RFP^negative^) (+367%, *P* < 0.0001) compared with macrophages (GFP^low^RFP^high^) (Fig. 1 *A* and *B*). Anti-CD47 led to significant influx of macrophages (+68%, *P* = 0.018) but not microglia [not significant (n.s.)] (Fig. 1*A* *Left* and *B*).

We recapitulated the experiment in *Ccr2* knockout mice to elucidate whether there was an increase of microglia in the absence of infiltrating peripheral macrophages upon anti-CD47 treatment. No significant changes were observed in microglial composition (Fig. 1 *A* and *C*). Even though *Ccr2* loss is known to prevent CNS infiltration by macrophages we observed a minor remaining GFP^low^ RFP^high^ population (Fig. 1 *A* and *C*).

### Anti-CD47 Treatment Induces Microglial Tumor Phagocytosis in a GBM Xenograft Model.

**Fig. 2.**
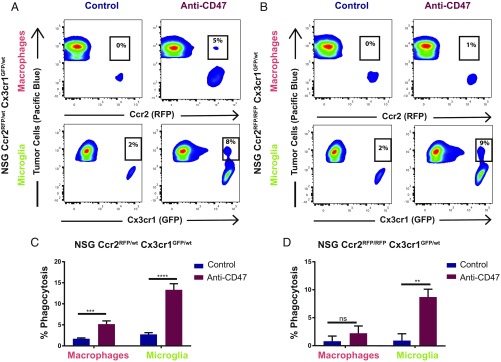
Anti-CD47 treatment enhances microglial tumor phagocytosis independent of macrophage recruitment. **(***A* and *B***)** Representative FACS plots of dissociated tumors from EBFP2^+^T387-grafted (*A*) NSG-*Ccr2*^*RFP/wt*^*Cx3cr1*^*GFP/wt*^ and (*B*) NSG-*Ccr2*^*RFP/RFP*^*Cx3cr1*^*GFP/wt*^ mice treated with anti-CD47 or control until they reached morbidity. Microglia were defined as GFP^high^RFP^negative^ and macrophages as RFP^high^GFP^low^. Anti-CD47 led to a significant increase of the double positive EBFP2^+^GFP^+^ microglial and EBFP2^+^RFP^+^ macrophage population in the *Ccr2*^*RFP/wt*^ mouse model. In the *Ccr2*^*RFP/RFP*^ model anti-CD47 treatment led to a significant increase of the EBFP2^+^GFP^+^ microglial population only. (*C* and *D*) Statistical analysis of the percentage of EBFP2^+^GFP^+^ microglia and EBFP2^+^RFP^+^ macrophages ± anti-CD47 treatment in (*C*) NSG-*Ccr2*^*RFP/wt*^*Cx3cr1*^*GFP/wt*^ and (*D*) NSG-*Ccr2*^*RFP/RFP*^*Cx3cr1*^*GFP/wt*^ mice after 3 wk of treatment. ***P = 0.0003; ****P < 0.0001; **P = 0.0019; Welch’s *t* test. Results are pooled from three independent experiments. (NSG-*Ccr2*^*RFP/wt*^*Cx3cr1*^*GFP/wt*^ control group *n* = 12, anti-CD47 group *n* = 17) (NSG-*Ccr2*^*RFP/RFP*^*Cx3cr1*^*GFP/wt*^ control group *n* = 7, anti-CD47 group *n* = 7). Mean ± SEM. ns, not significant.

To ascertain if TA-MG can act as effector cells upon CD47-SIRPα blockade, we analyzed the frequency of in vivo phagocytized tumor cells 3 wk after anti-CD47 or control treatment by flow cytometry. Indeed, anti-CD47 treatment induced a significant increase of in vivo tumor cell phagocytosis by TA-MG (2.7% control versus 13.3% treated, *P* < 0.0001) as assessed by the presence of GFP^high^RFP^negative^EBFP2^+^ microglia (Fig. 2 *A* and *C*). We also observed enhancement of tumor cell phagocytosis by TA-MAC (1.7% control versus 5.2% treated, *P* = 0.0003) defined by RFP^high^GFP^low^EBFP2^+^ macrophages (Fig. 2 *A* and *C*).

Despite the presence of a minor TA-MAC population in CCR2 knockout mice, we found no significant tumor cell phagocytosis of TA-MAC (Fig. 2 *B* and *D*). In contrast, tumor cell phagocytosis by microglia was significantly enhanced upon anti-CD47 treatment (0.9% control versus 8.7% treated, *P* = 0.0019) (Fig. 2 *B* and *D*). These results indicate that resident microglia are independently responsive to CD47-SIRPα blockade.

### Anti-CD47 Immunotherapy Induces Microglia-Mediated Survival Benefit.

**Fig. 3.**
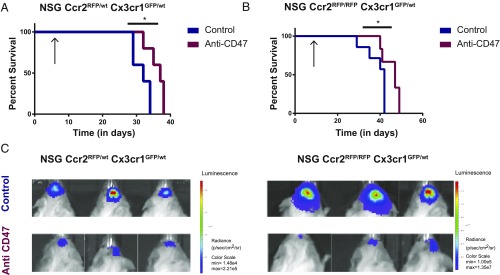
Anti-CD47 treatment leads to significant survival benefit in T387 grafted mice even in absence of phagocytizing macrophages. (*A* and *B*) Kaplan–Meier curves of (*A*) NSG-*Ccr2*^*RFP/wt*^*Cx3cr1*^*GFP/wt*^ (*P = 0.01) and (*B*) *Ccr2*^*RFP/RFP*^*Cx3cr1*^*GFP/wt*^ mice (*P = 0.049) grafted with T387-EBFP2-luc treated with anti-CD47 (H5F9-G4) or human IgG control; purple, anti-CD47 treated; blue, control (human IgG). Arrows indicate beginning of treatment on day 7 after tumor injections. (*C*) Images of luminescence measurements detected with an IVIS spectrum instrument on day 30 after tumor injections. Minimum and maximum values were adjusted to the same level for the NSG-*Ccr2*^*RFP/wt*^*Cx3cr1*^*GFP/wt*^ and *Ccr2*^*RFP/RFP*^*Cx3cr1*^*GFP/wt*^ model. Representative survival experiment is shown. (NSG-*Ccr2*^*RFP/wt*^*Cx3cr1*^*GFP/wt*^ control group *n* = 5, anti-CD47 group *n* = 5) (*Ccr2*^*RFP/RFP*^*Cx3cr1*^*GFP/wt*^ control group *n* = 7, anti-CD47 group *n* = 6). Mean ± SEM.

We next wanted to investigate if microglia alone can mediate efficacy against GBM xenografts upon anti-CD47 treatment. After orthotopic injection of T387 tumor cells into either NSG-*Ccr2*^*RFP/wt*^*-Cx3cr1*^*GFP/wt*^ or NSG-*Ccr2*^*RFP/RFP*^*-Cx3cr1*^*GFP/wt*^ mice and confirmation of tumor engraftment via bioluminescence imaging we started treatment with anti-CD47 (250 µg Hu5F9-G4 three times a week) or human IgG control. We observed significant survival benefit in the *Ccr2* haploinsufficient mice when treated with anti-CD47 compared with control (Fig. 3 *A* and *C*).

As expected in the *Ccr2* knockout mice we observed a delay in tumor-induced morbidity presumably due to the lack of tumor supporting macrophages (Fig. 3*B*) (15). Importantly, anti-CD47 treatment led to a similar survival benefit in the absence of *Ccr2*, suggesting an effector role for microglia in myeloid checkpoint inhibition even in the absence of peripheral macrophages (Fig. 3 *B* and *C*). To rule out direct inhibitory effects of anti-CD47 on glioma cells, we performed an in vitro proliferation assay and found no impairment of cell growth upon anti-CD47 treatment in two different glioma lines (T387, *P* = n.s.; T3832, *P* = n.s.), whereas the same concentration of anti-CD47 led to a highly significant increase of in vitro phagocytosis of four different glioma lines when cocultured with bone marrow-derived macrophages of NSG-*Ccr2*^*RFP/wt*^*-Cx3cr1*^*GFP/wt*^ (T387, *P* = 0.003; T4121, *P* < 0.0001; T3832, *P* = 0.002; and T3691, *P* = 0.002) (*SI Appendix*, Fig. S4).

**Fig. 4.**
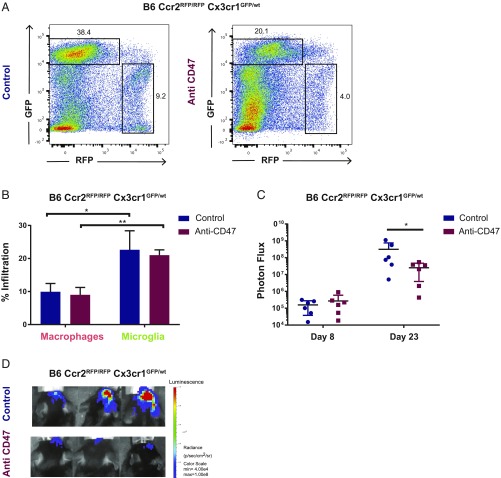
Anti-CD47 treatment leads to macrophage-independent survival benefit in a syngeneic glioma model. (*A*) Representative FACS plots of the TAM pool in anti-CD47 treated and control condition in CT-2A-luc grafted B6-*Ccr2*^*RFP/RFP*^*Cx3cr1*^*GFP/wt*^ mice, gated on CD45 positive cells. (*B*) Composition of the TAM pool in CT-2A-luc grafted B6-*Ccr2*^*RFP/RFP*^*Cx3cr1*^*GFP/wt*^ mice as assessed by flow cytometry analyses on GFP^low^RFP^+^ (macrophages) and RFP^negative^GFP^bright^ (microglia) signal. Expressed as a percent of CD45 positive cells. *P = 0.041; **P = 0.003. (control group *n* = 5, anti-CD47 group *n* = 5) (*C*) Luminescence measurements (photon flux in p/s) for CT-2A-luc grafted B6-*Ccr2*^*RFP/RFP*^*Cx3cr1*^*GFP/wt*^ mice treated with anti-CD47 (MIAP401) or mouse IgG control. *P = 0.041, (control group *n* = 6, anti-CD47 group *n* = 6) Mann–Whitney *U* test. (*D*) Representative images of luminescence measurements detected with an IVIS spectrum instrument on day 23 after tumor injections. Mean ± SEM.

We further validated our findings in a syngeneic glioma model using the mouse glioma cell line CT2A-LUC. In the absence of *Ccr2* the TAM pool of B6-*Ccr2*^*RFP/RFP*^*-Cx3cr1*^*GFP/wt*^ mice was highly dominated by microglia (+133%, *P* = 0.003) (Fig. 4 *A* and *B*). As observed in the immunocompromised xenograft model, no increase in microglia composition was observed upon anti-CD47 treatment (Fig. 4 *A* and *B*). Similarly, even in the absence of a functioning Ccr2 receptor, inhibiting peripheral macrophage infiltration, anti-CD47 treatment was efficacious and reduced tumor burden significantly as indicated by bioluminescence (Fig. 4 *C* and *D*). These results confirm our previous findings that microglia themselves mediate important effector functions upon myeloid checkpoint blockade in an intact immune system.

### In Vivo Visualization of Microglial Phagocytosis upon Anti-CD47 Treatment.

**Fig. 5.**
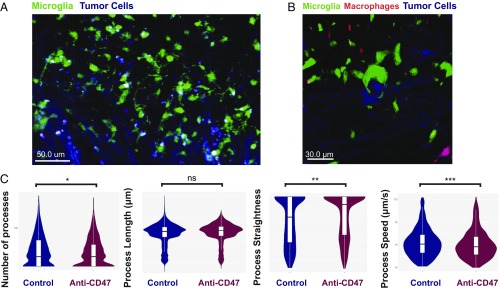
In vivo imaging shows real-time phagocytosis of living glioma cells by microglia and morphologic changes upon anti-CD47 treatment. (*A* and *B*) Two-photon in vivo laser scanning image of T387-EBFP2^+^ grafted NSG-Ccr2^RFP/wt^Cx3cr1^GFP/wt^ mice after 2 wk of anti-CD47 treatment. Tumors were implanted at a depth of 2 mm below the right frontal cortex and cranial windows were implanted; microglia, green; tumor cells, blue; and macrophages, red. (*C*) Violin plots of microglial filament tracking parameters using binomial logistic regression for anti-CD47 treated or control condition (based on corresponding Movies S6 and S7). Multivariate logistic regression identified an increase of number of processes (*P = 0.046) and process straightness (**P = 0.0057) in anti-CD47 treated cells, whereas process speed decreased significantly (***P = 0.005). Process length was not significantly different between conditions [*P* = not significant (ns)].

To have a better understanding of the effect of anti-CD47 treatment on the morphology and real-time kinetics of microglia response, we performed intracranial in vivo imaging of NSG-*Ccr2*^*RFP/wt*^*-Cx3cr1*^*GFP/wt*^ mice grafted with the tumor cell line T387-EBFP2^+^-Luc. In accordance with our flow cytometry results, we observed a remarkable recruitment of macrophages upon anti-CD47 treatment (+68%, *P* = 0.018) (Fig. 1*B* and Movies S1 and S2). Still, TA-MG clearly outweighed TA-MAC in numbers (+117%, *P* < 0.0001) (Fig. 1 *A* and *B*). Strikingly, we could capture a high proportion of microglia phagocytizing living tumor cells in real time upon anti-CD47 treatment (Fig. 5 *A* and *B* and Movies S3 and S4). On the other hand, in the control situation, single tumor cells were only surveilled by microglial processes, but we were unable to visualize phagocytosis (Movie S5). To examine the morphological changes associated with anti-CD47 treatment, we compared TA-MG from mice treated with anti-CD47 or control and quantified microglial process movement using filament tracking algorithms. Interestingly, the number of microglial processes (+39.9%, *P* = 0.04) and straightness significantly increased (+241.0%, *P* = 0.005) upon anti-CD47 treatment (Fig. 5*C* and Movie S6 and S7), whereas the mean speed of the process movement was significantly decreased in the treated condition. This suggests a more active and directed filament movement as well as a prolonged interaction time with tumor cells.

### Distinct Transcriptional Profile of Microglia Versus Macrophages in Response to Anti-CD47 Immunotherapy.

**Fig. 6.**
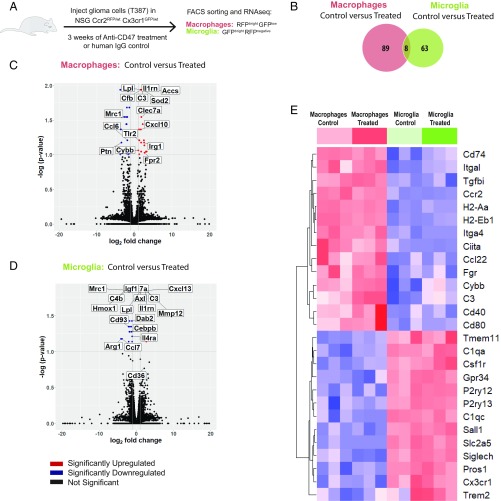
RNA-seq analysis reveals distinct transcriptional profiles of tumor-associated macrophages and microglia. (*A*) Experimental scheme for the RNA-seq experiment. (*B*) Venn diagram of significantly differentially expressed genes (*q* value <0.1) in TA-MAC (red) (control group *n* = 3, anti-CD47 group *n* = 3) and TA-MG (green) (control group *n* = 3, anti-CD47 group *n* = 3) of mice treated with anti-CD47 or control. (*C* and *D*) Volcano plots of differentially expressed genes between (*C*) TA-MAC control versus treated and (*D*) TA-MG control versus treated. The *P* values are Benjamini–Hochberg corrected. (*E*) Heatmap depicting row *z* scores of log_2_-transformed gene expression of TA-MAC control (light red), TA-MAC treated (red), TA-MG control (light green), and TA-MG treated (green).

Given their differential lineage origins, we investigated whether the functional and morphological changes of TA-MG and TA-MAC upon anti-CD47 treatment were also associated with distinctive gene expression patterns. For this study, TA-MG (GFP^high^RFP^negative^) and TA-MAC (GFP^low^RFP^high^) from T387-EBFP-Luc grafted NSG-*Ccr2*^*RFP/wt*^*-Cx3cr1*^*GFP/wt*^ mice were sorted and processed for RNA sequencing after 3 wk of either anti-CD47 or control treatment (Fig. 6*A*).

Consistent with a previous study, compared with TA-MG, we found TA-MAC to express higher levels of genes associated with the alternative complement system (*Cfb*, *Cfp*), antigen-presenting functions (*Cd40*, *Cd80*), immune receptors and signaling (*Tlr8*, *Jak2*), and chemotaxis (*Ccl17*, *Ccl22*) (Fig. 6*E*) (*SI Appendix*, Fig. S2*D*). We submitted the differentially expressed genes (*q* value <0.05) between TA-MG and TA-MAC to Ingenuity Pathway Analysis (IPA) for functional analysis. Interestingly, functions including “inflammatory response” (*z* score = 4.3, *q* value = 1.75 × 10^−7^), “cell movement of myeloid cells” (*z* score = 2.8, *q* value = 1.2 × 10^−6^), “activation of antigen-presenting cells” (*z* score = 2.6, *q* = 9.34 × 10^−5^), and “chemotaxis of lymphocytes” (*z* score = 2.9, *q* value = 1.48 × 10^−6^) were significantly more enriched in TA-MAC than in TA-MG (*SI Appendix*, Fig. S2*C*).

Upon anti-CD47 treatment we found 63 genes to be differentially expressed by TA-MG and 89 genes by TA-MAC (Fig. 6*B*). Intriguingly, only eight genes were differentially expressed by both TA-MG and TA-MAC (Fig. 6*B*). This indicates that CD47 blockade leads to a distinct response pattern between TA-MG and TA-MAC. TA-MAC showed a significant up-regulation (*q* value <0.1, fold change >2) of immune receptors, chemokines (*Il1rn*, *Cxcl10*, *Tlr2*), complement factors (*C3*, *Cfb*), reactive oxygen species (*Cybb*, *Sod2*), M1 markers (*Fpr2*, *Irg1*), and the down-regulation of the M2 marker *Mrc1*, suggesting a notable proinflammatory response (Fig. 6*C*). TA-MG on the other hand down-regulated immune receptor-associated genes (*Hmox1*, *Il4ra*, *Ccl7*) and showed an increased expression level of antiinflammatory genes (*Dab2*, *Mmp12*), suggesting a less inflammatory reaction (Fig. 6*D*). Intriguingly, genes associated with the phagocytic machinery (*Axl*) were only up-regulated in TA-MG (Fig. 6*D*).

## Discussion

TAMs are a major immune component of many types of cancer and can account for up to 50% of cells within the brain tumor microenvironment. Monocyte-derived macrophages are recruited by release of cytokines and chemokines due to tissue perturbation (5). While brain resident microglia are critical regulators of tissue homeostasis, their role in immune surveillance of glioma cells remains poorly understood. The evasion of programmed cell death and subsequent programmed cell removal is one of several key early events that need to be overcome in the progression from normal cellular homeostasis to malignant transformation (16). Brain cancer cells employ multiple strategies to evade innate and adaptive immunity. While we have previously shown that blocking the myeloid checkpoint CD47-SIRPα axis is efficacious against multiple adult and pediatric brain tumors, the specific role of microglia in anti-CD47 treatment remains unclear (9, 10, 17, 18).

To delineate the GBM TAM pool we utilized a model with genetically color-coded macrophages (*Ccr2*^RFP^) and microglia (*Cx3cr1*^GFP^), which allowed us to distinguish resident microglia from infiltrating, blood-derived macrophages. The TAM composition of GBM was dominated by microglia in all tumor models. Investigating the effect of the myeloid checkpoint inhibitor anti-CD47 we found that macrophages and microglia respond by phagocytizing tumor cells. Even in the absence of infiltrating macrophages (*Ccr2*^RFP/RFP^), microglia continue to phagocytize tumor cells resulting in a significant survival benefit. RNA-seq analysis showed the enrichment of microglia-specific markers such as *Tmem119*, *Sall1*, and *Pros1* as well as macrophage-specific markers such as *Itga4* and *Itg1* validating the differentiation between TA-MG and TA-MAC in our model (12, 19, 20). Importantly, the transcriptional profile of TA-MG was characterized by a less inflammatory response to anti-CD47 treatment making the reeducation of microglia a clinically attractive target.

Most therapies targeting the tumor microenvironment focus on the depletion of the TAM pool to suppress their protumorigenic effect. However, reeducating TAMs has the additional benefit of an active targeting of tumor cells (21). This is of particular interest since TAMs are genetically stable and more resistant to treatment-associated mutations (22). TAM reeducation with anti-CD47 is especially promising as SIRPα, the receptor of CD47, is exclusively expressed on myeloid cells. CD47 is ubiquitously overexpressed on malignant human tumors, including malignant pediatric brain tumors and GBM (9, 10). The clinical relevance of immune escape by CD47 up-regulation on tumor cells is also reflected by correlation studies: high levels of CD47 expression are associated with a decreased probability of overall survival in GBM (9).

Treatment-associated neurotoxicity is a common problem of immunotherapy. Cytokine release causes inflammation of the brain which is associated with serious complications like cerebral edema and increase of intracranial pressure (23, 24). In this context it might be especially important that we find microglia showing a less inflammatory response to anti-CD47, although the phagocytosis rate of TA-MG is even higher than that of TA-MAC. This makes microglia an attractive target for clinical applications.

Triggering of phagocytosis is a balancing act determined by pro- and antiphagocytic signals. The blockade of CD47 is only effective in combination with the presence of prophagocytic signals such as calreticulin and phosphatidylserine. A limitation of our study is the modest efficacy of anti-CD47 treatment alone. Future studies combining anti-CD47 therapy with cytotoxic therapies such as chemotherapy and low-dose radiation, which are known to increase surface expression of prophagocytic signals, need to be conducted to assess the full potential of CD47 blockade (25). Other studies demonstrated that anti-CD47 therapy elicits a type I IFN-mediated priming of the adaptive immune system (26). Thus, combinational therapies with checkpoint inhibitors and other regulators of the adaptive immune system might also be promising. This is also supported by a recent report of a phase Ib/II study with non-Hodgkin lymphoma patients showing encouraging clinical responses for the combination therapy of anti-CD47 and rituximab (27).

To date only few options exist to selectively deplete macrophages. Pharmacological depletion of brain tumor-associated macrophages with either anti-CSF1R antibodies or liposomal clodronate is effective but unselective, since it also depletes microglia (28, 29). Homozygous knockout of CCR2 prevents selectively the entry of monocytes into the CNS while maintaining microglial function. The absence of T, B, and NK cells in NSG mice enables a more precise analysis of myeloid cell function without confounding influence by the adaptive immune system. We further ruled out direct inhibitory effects of anti-CD47 as another potential underlying antitumor effect on glioma cells as suggested by a previous study by Li et al. (30) and found no impairment of tumor cell proliferation by anti-CD47 treatment. Our analysis of myeloid infiltration demonstrated that a small number of monocytes is able to penetrate the blood brain barrier (BBB) independent of Ccr2. This might be due to the disrupted BBB that is often encountered in malignant brain tumors (31). However, these infiltrating macrophages were not capable of phagocytosis so that we could demonstrate a macrophage-independent antitumor effect by microglia. This effect was maintained in our syngeneic glioma model indicating that interactions with the adaptive immune system do not impede the reeducation of microglia. The antibodies used in each of the cases have significantly different binding affinities. The anti-HuCD47 mAb has a 100-fold higher binding affinity compared with the anti-msCD47 mAb hence showing different levels of efficacy. Furthermore the NOD-*SIRPα* gene exhibits high polymorphism which enables high-affinity binding to human CD47 which has very low binding affinity to WT B6-*SIPRα*. In summary, our data demonstrate the distinct reactions of TA-MG and TA-MAC to anti-CD47 treatment. We show in vivo that microglia are able to effectively phagocytize glioma cells. Blocking the myeloid checkpoint-inhibitor CD47 enhances the phagocytosis rate, resulting in a significant survival benefit even in the absence of peripheral macrophages. Our study shows an immunomodulatory treatment that reeducates microglia to target tumor cells directly.

## Materials and Methods

### Cell Lines and Culture.

The T387, T3832, 4121, and 3691 cell lines were generously provided by Jeremy Rich, Cleveland Clinic, Cleveland, OH, and grown in serum-free media. The mouse glioma cell line CT-2A was provided by Thomas Seyfried, Boston College, Boston and maintained in media consisting of DMEM, 10% FBS, penicillin/streptomycin, and GlutaMAX (*SI Appendix*, Table S1). Cell lines were infected with EF1-EBFP2-T2A-Luciferase lentivirus, double-sorted for EBFP2 signal, and grown in spheres in defined media.

### Mice.

All mice were housed in specific pathogen-free conditions at a barrier facility at the Lokey Stem Cell Building (SIM1) at Stanford University School of Medicine. All animal handling, surveillance, and experimentation was performed in accordance with and approval from the Stanford University Administrative Panel on Laboratory Animal Care.

### Generation of NSG-Ccr2^RFP/wt^Cx3cr1^GFP/wt^ and NSG-Ccr2^RFP/RFP^Cx3cr1^GFP/wt^ Mice.

*Ccr2*^*RFP/RFP*^ mice (13) and *Cx3cr1*^*GFP/GFP*^ mice (32) on a C57BL/6 background were purchased from The Jackson Laboratory and intercrossed to yield Ccr2^*RFP/wt*^*-Cx3cr1*^*GFP/wt*^ animals. Double heterozygous animals were backcrossed to *Ccr2*^*RFP/RFP*^ mice to obtain Ccr2^*RFP/RFP*^*-Cx3cr1*^*GFP/wt*^ animals. Further, male B6-*Ccr2*^*RFP/RFP*^ and *Cx3cr1*^*GFP/GFP*^ mice were mated with female *NSG* (*NOD.Cg-Prkdc*^*scid*^ *Il2rg*^*tm1Wjl*^*/SzJ*, lacking T, B, NK cells) mice (33). The heterozygous males of the first generation carrying the X-chromosomal *Il2rg* mutation were used for the next backcrossing to female *NSG* mice. This crossing yielded a small number of *Ccr2*^*RFP/wt*^ and *Cx3cr1*^*GFP/wt*^*Il2rg*^*−/−*^*Prkdc*^*scid/scid*^*Sirpα*^*NOD/NOD*^ mice. The positive mice were intercrossed to yield *NSG-Ccr2*^*RFP/RFP*^ and *NSG-Cx3cr1*^*GFP/GFP*^ and resulting offsprings were intercrossed once more to generate the target animals. Based on these breedings, both single fluorescent *NSG-Cx3cr1*^*GFP/wt*^ and *NSG-Ccr2*^*RFP/wt*^ animals as well as animals carrying the combination *NSG-Cx3cr1*^*GFP/wt*^-*NSG-Ccr2*^*RFP/RFP*^ with deficient Ccr2 signaling were obtained. Primers (in 5′–3′ direction) used for genotyping with resulting amplification products are listed in *SI Appendix*, Table S2. Genotyping of the *Prkdc*^*Scid*^ mutation was done as previously described (34). A representative agarose gel electrophoresis plot after PCR amplification is shown in *SI Appendix*, Fig. S1*B*.

*Sirpα* polymorphism has been shown to be of high importance for successful xenografting (35). To discriminate between NOD and C57BL/6 *Sirp*α, a restriction fragment length polymorphism analysis was established. Briefly, *Sirp*α exon 2 was sequenced, restriction sites mapped, PCR amplified, and the resulting fragment digested with restriction enzyme Bsh1236I (FastDigest, Thermo Fisher), which specifically cuts the NOD-*Sirpα* amplicon into two fragments (290 bp/112 bp), whereas the C57BL/6 *Sirp*α amplicon remains undigested. Further, absence of T, B, and NK cells in established *NSG-Ccr2*^*RFP/RFP*^ mice was verified by FACS analysis of PBMCs from *NSG-Ccr2*^*RFP/RFP*^ and NSG control mice using antibodies against CD3, CD19, and NK1.1 (all BioLegend) (*SI Appendix*, Table S2).

### Orthotopic Xenograft Model for Brain Tumors.

T387-EBFP2-Luc cells were dissociated to single cells and orthotopically injected into 6- to 10-wk-old *NSG-Ccr2*^*RFP/wt*^*Cx3cr1*^*GFP/wt*^ and *Ccr2*^*RFP/RFP*^*Cx3cr1*^*GFP/wt*^ mice. CT-2A-Luc cells were injected into 6- to 10-wk-old B6-*Ccr2*^*RFP/wt*^*Cx3cr1*^*GFP/wt*^ and *Ccr2*^*RFP/RFP*^*Cx3cr1*^*GFP/wt*^ mice. In brief, mice were anesthetized with 3% isoflurane (Minrad International) in an induction chamber. Anesthesia on the stereotactic frame (David Kopf Instruments) was maintained at 2% isoflurane delivered through a nose adaptor. A burr hole was placed 2 mm lateral and 2 mm posterior of bregma. A blunt-ended needle (75N, 26s/2-inch point style 2, 5 μL; Hamilton Co.) was lowered into the burr hole to a depth of 3 mm below the dura surface and retracted 0.5 mm to form a small reservoir. Using a microinjection pump (UMP-3, World Precision Instruments), 1 × 10^5^ CT-2A-Luc cells or 1× 10^5^ T387-EBFP2-Luc cells were injected in a volume of 3 µL at 30 nL/s. After leaving the needle in place for 1 min, it was retracted at 3 mm/min.

Tumor formation was followed by bioluminescence imaging on an IVIS spectrum instrument (Caliper Life Science) and quantified with Live Image 4.0 software (Living Image, PerkinElmer). T387-EBFP2-luc engrafted mice were treated with i.p. injections of 250 µg anti-CD47 antibody (H5F9-G4) or human IgG control three times a week (Monday/Wednesday/Friday) until they reached morbidity. CT-2A-luc grafted mice were treated with 250 µg of anti-CD47 antibody (MIAP401) or mouse IgG control. All in vivo experiments were conducted at least two times unless mentioned otherwise.

### Flow Cytometry Analysis and Cell Sorting.

Mice were deeply anesthetized with ketamine/xylazine (87.5 mg/kg ketamine, 12.5 mg/kg xylazine) and transcardially perfused with ice-cold PBS. Brains were removed and the tumors were either macroscopically dissected or by using a fluorescent microscope. Specimens were minced in RPMI, enzymatically dissociated at 37 °C for 10 min with 250 units/mL DNase1 (Worthington) in a solution containing Hank’s balanced salt solution (HBSS) with Ca^2+/^Mg^2+^, nonessential amino acids, sodium pyruvate, Hepes, glutamine and antibiotic-antimycotic (Corning). Following mechanical trituration, cells were filtered through 70-µm and 40-µm filters and subjected to a 0.9 M sucrose gradient centrifugation to dispose of dead cells and myelin. Upon ACK lysis for removal of erythrocytes (Gibco Life Technologies) cells were resuspended in FACS buffer containing HBSS, 1% BSA, nonessential amino acids, sodium pyruvate, Hepes, EDTA, glutamine, and antibiotic-antimycotic (Corning) and stained with the following antibodies: Antibodies used for flow cytometry analysis are shown in *SI Appendix*, Table S3. DAPI stain (Invitrogen) or Sytox Red (APC, Invitrogen) were used to exclude dead cells.

Flow cytometric analysis and cell sorting were performed on the BD FACS Aria II (Becton Dickinson). The flow-cytometry gating strategy for sorting of microglia and macrophages is depicted in *SI Appendix*, Fig. S5.

### Cranial Window Surgery.

At 5 d before the implantation of the windows, orthotopic tumors (1 × 10^5^ T387-EBFP2-Luc cells) were transplanted at a depth of 2 mm below the dura as described above. Eight- to 12-wk-old NSG-*Ccr2*^*RFP/wt*^*-Cx3cr1*^*GFP/wt*^ mice were anesthetized via intramuscular injection with ketamine/xylazine (87.5 mg/kg ketamine, 12.5 mg/kg xylazine). The animals’ body temperatures were maintained at 36.5–37.5 °C using a heating pad connected to a controller (DC temperature controller, FHC), and their eyes were covered with eye protecting gel (Solcorin; Solco Basie, Ltd). The scalp was shaved and the surgical field scrubbed and thoroughly disinfected with ethanol and betadine. Thereafter, the frontobiparietal scalp and periosteum was excised and surface of the cranium irrigated with hydrogen peroxide. A right-sided or bilateral frontoparietal bone flap was drilled and carefully detached from the underlying dura mater followed by extensive irrigation with 0.9% saline. We opened and removed the dura on the right hemisphere with a thin 30-G needle under the microscope under strict avoidance of superior sagittal sinus or other venous bleeding. We irrigated again with saline and covered the tumor-centered craniectomy with a sterile, thick cover glass (hemocytometer coverglass, VWR) that was previously size adjusted using a diamond cutter. The borders of the coverglass were glued to the bone using cyanoacrylate glue (Loctite). For recovery, mice were s.c. injected with 0.1 mg/kg buprenorphine and fed with antibiotic-enriched UNIPRIM food (Neogen). Subsequently, mice were treated with 250 µg of anti-CD47 antibody (H5F9-G4) or human IgG control three times a week (Monday/Wednesday/Friday) beginning at day 7 after tumor injections. Tumor engraftment was verified by bioluminescence imaging before treatment began.

### Two-Photon In Vivo Microscopy and Confocal in Vivo Microscopy.

Intravital two-photon images were acquired with a Prairie Ultima IV two-photon in vivo microscope (Bruker) equipped with two Ti:Sapphire lasers (MaiTai HP, Spectra Physics) tuned to 810 nm (for excitation of EBFP2^+^ tumor cells) and 920 nm (for excitation of GFP^+^ microglia and RFP^+^ macrophages), respectively, and focused through an Olympus XLUM Plan Fluor 20× water immersion lens (numeric aperture of 0.95). Before imaging, mice were anesthetized with isoflurane and the head was fixed in a stereotactic frame (Kopf Instruments). Anesthesia was maintained at 1% isoflurane through a nose cone, and body temperature was kept stable via a temperature-controlled heating pad. Images of 598 × 598 µm^2^ areas of in vivo tumors were acquired at 1,024 × 1,024 pixel resolution (0.584 µm per pixel). Volume images were acquired over a 100- to 200-µm Z range in 1-µm steps. Time-lapse datasets were acquired either in single planes over time periods up to 90 min, or in combined time + Z series over a 598 × 598 × 7 µm^3^ volume (X × Y × Z). Movies were processed in ImageJ software; for 3D reconstructions, data were imported into Imaris software (BitPlane) for surface rendering. Microglial process movement over time was quantified using the filament tracking tool of Imaris. Quantitative parameters were extracted and utilized for univariate and multivariate logistic regression, with anti-CD47 treatment serving as a binomial response variable.

Confocal in vivo microscopy was performed using an Olympus IV100 intravital laser scanning microscope. To visualize blood vessels, AngioSense 680 (PerkinElmer) dye was injected into the tail vein. Data were analyzed with ImageJ and Imaris.

### RNA Extraction from Sorted Cells, cDNA, and Library Preparation.

After grafting *NSG-Ccr2*^*RFP/wt*^*Cx3cr1*^*GFP/wt*^ mice with the T387-EBFP2-luc cell line and treating the mice for 3 wk with i.p. injections of 250 µg of anti-CD47 (H5F9-G4) or human IgG control three times a week (Monday/Wednesday/Friday), we sorted between 2,000 and 30,000 tumor-associated microglia and macrophages directly into TRIzol LS (Invitrogen). RNA was extracted via a combined TRIzol and column cleanup protocol for low cell amounts. Additionally, the RNA was DNase treated. Subsequently, the integrity of extracted RNA was assayed by on-chip electrophoresis (Agilent Bioanalyzer) and only samples with a high RNA integrity (RIN) value >7 were used for RNA-seq. Purified total RNA was reverse transcribed into cDNA using the Ovation RNA-seq System V2 (NuGEN) and cDNA was sheared using the Covaris S2 system (duty cycle 10%, intensity 5, cycle/burst 100, total time 5 min). Sheared cDNA was cleaned up using Agencourt AMPure XP beads (Beckman Coulter) and ligated to adaptors (Illumina) Sequencing libraries were constructed using the NEBNext Ultra DNA Library Prep Kit (New England Biolabs) using barcoded adaptors to enable multiplexing of libraries on the same sequencing lane. For each RNA-seq library, the effectiveness of adaptor ligation and effective library concentration was determined by Bioanalyzer before loading them in multiplexed fashion onto an Illumina NextSeq500 (Stanford Functional Genomics Facility) to obtain 150 bp paired-end reads.

### RNA-Seq Analysis.

First, raw reads were trimmed using Skewer (v0.2.2) to remove the adaptors. We then followed the ENCODE long RNA-seq pipeline for expression quantification (https://www.encodeproject.org/rna-seq/long-rnas/) (The ENCODE Project Consortium, 2012). Specifically, reads were aligned to the mm10 genome with STAR (v2.5.3) using two-pass mapping (36). Genome indices were created with Samtools (v1.4.1) (37). For expression level quantification and differential expression analysis, we used the Cufflink package (v2.2.1) and applied the Cuffdiff and Cuffquant programs to the aligned reads (37). For functional analysis with IPA (v01–10) we provided fragments per kilobase million (FPKM), log_2_(fold change), *P* values, and false discovery rates from Cufflink.

### Proliferation Assay.

For real-time analysis, IncuCyte Zoom System (Essen BioScience) was utilized. T387 or T3832-RFP cells were seeded into 96-well plates and treated with a final concentration of 10 μg/mL anti-CD47 or PBS. The culture plate was incubated in an IncuCyte Zoom System in a cell culture incubator. Cell images were captured in four fields per well every 3 h. Total red object integrated intensity (GCU × µm2/image) was determined using IncuCyte Zoom software (Essen BioScience).

### In Vitro Phagocytosis Assay.

Mouse macrophages were obtained from 7- to 10-wk-old NSG-*Ccr2*^*RFP/wt*^ mouse bone marrow. Briefly, mice were killed in a CO_2_ chamber and femura and tibiae were isolated. The bones were kept in ice-cold PBS and sterilized in 70% ethanol. By flushing them with mouse macrophage medium (RPMI with 10% FBS, 1× penicillin/streptomycin, 200 mM glutamine, and 25 mM Hepes, all from Corning, Inc.), bone marrow cells were gathered and plated at 1 × 10^6^/mL in 100 × 25 mm Petri dishes in mouse macrophage medium containg 10 nM m-CSF. The medium was changed after 3 d and cells were harvested for experiments on days 7–10.

Dissociated EBFP2^+^ tumor cells were incubated with bone marrow-derived macrophages in serum-free Iscove’s modified Dulbecco minimum essential medium with or without prior addition of 10 µg/mL anti-CD47 monoclonal antibody (H5F9-G4, IgG4) at 37 °C for 2 h in ultra-low attachment round-bottom 96-well plates. The tumor cell to macrophage or microglia ratio was 2:1. Cells were analyzed with a BD LSR Fortessa (Becton Dickinson) using a high throughput sampler. Gates were placed according to unstained and fluorescence-minus-one (FMO) controls. Technical duplicates of each experimental condition were obtained.

### Statistical Analysis.

All statistical analyses were performed using GraphPad Prism 7 software. Results were expressed as mean ± SEM. Welch’s *t* test was used for group comparisons (two tailed). Survival analysis was performed using a log-rank test. *P* < 0.05 was considered significant if not stated otherwise.

## Supplementary Material

Supplementary File

Supplementary File

Supplementary File

Supplementary File

Supplementary File

Supplementary File

Supplementary File

Supplementary File

Supplementary File
