# Supplementary material for: Microglia are effector cells of CD47-SIRPα antiphagocytic axis disruption against glioblastoma
Source: Proc Natl Acad Sci U S A. 2019 Jan 2;116(3):997–1006. doi: 10.1073/pnas.1721434116 (PMC6338872; doi:10.1073/pnas.1721434116)
Supplement: Supplementary File [file pnas.1721434116.sapp.pdf]

PNAS
www.pnas.org

Supplementary Information for

Microglia are effector cells of CD47-SIRPa anti-phagocytic axis disruption against glioblastoma

Gregor Hutter, Johanna Theruvath, Claus Moritz Graef, Michael Zhang, Matthew Kenneth Schoen, Eva Maria Manz, Mariko L. Bennett, Andrew Olson, Tej D. Azad, Rahul Sinha, Carmel Chan, Suzana Assad Kahn, Sharareh Gholamin, Christy Wilson, Gerald Grant, Joy Q. He, Irving L. Weissman, Siddhartha S. Mitra, Samuel H. Cheshier

Corresponding Authors:
Irving L. Weissman
E-mail: irv@stanford.edu
Siddhartha S. Mitra.
E-mail: siddhartha.mitra@ucdenver.
Samuel H. Cheshier
E-mail: samuel.cheshier@hsc.utah.edu

This PDF file includes:

Figs. S1 to S4
Captions for Movies S1 to S8
Table 1 to Table 3

Other supplementary materials for this manuscript include the following:

Movies S1 to S8

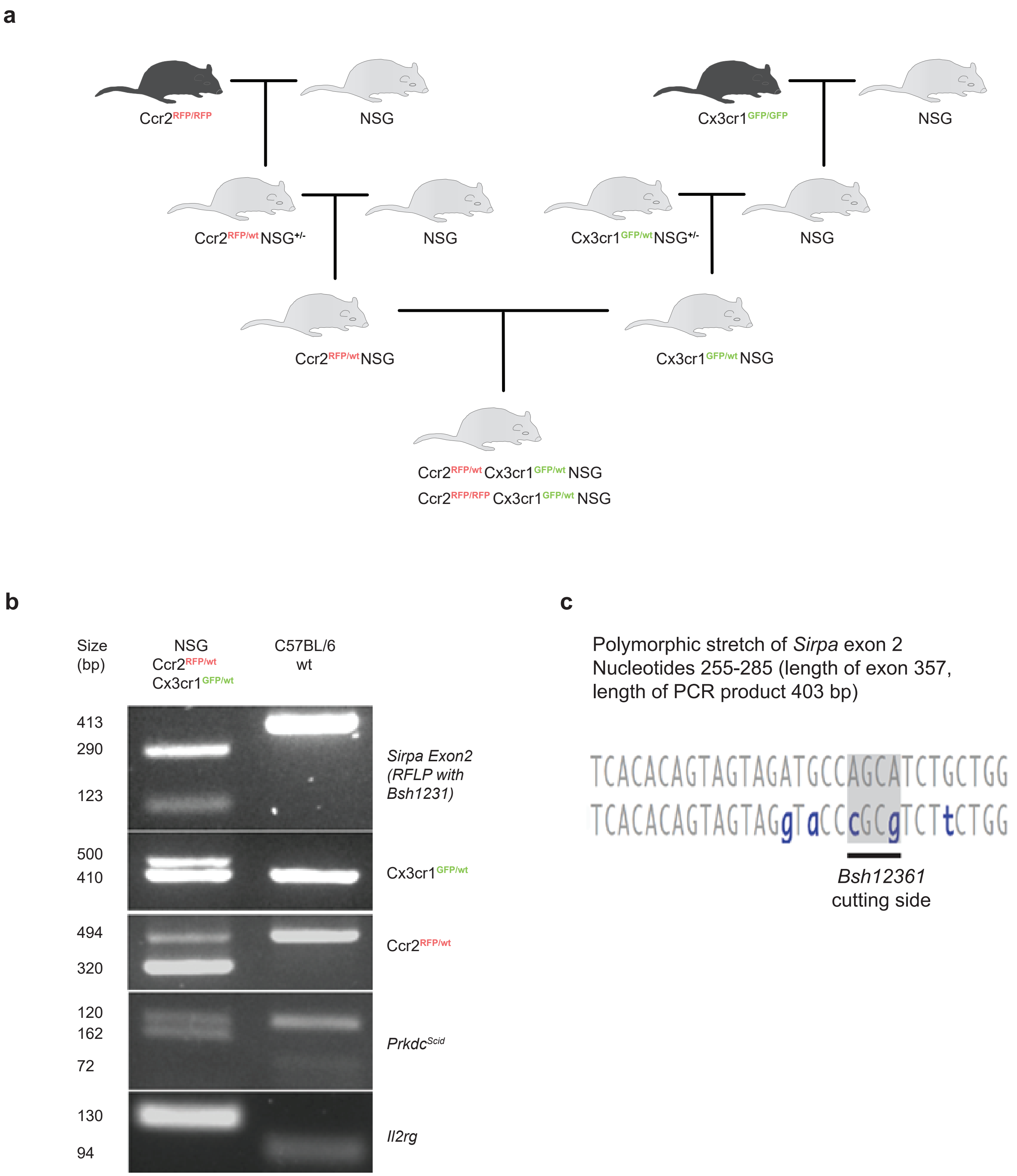

Supplementary Figure 1: Mouse breeding for NSG-Ccr2RFP/wt Cx3cr1GFP/wt and NSG-Ccr2RFP/RFP Cx3cr1GFP/wt mice
(a) Breeding scheme to yield desired NSG-Ccr2RFP/wtCx3cr1GFP/wt and NSG-Ccr2RFP/RFPCx3cr1GFP/wt via backcrossing of naive NSG mice with B6 Ccr2RFP/RFP and Cx3cr1GFP/GFP mice. (b) Representative results of genotyping PCRs loaded on 2% agarose gel reflecting the allelic status of the involved mutated genes (Sirpa, Il2rg, Prkdcscid, Cx3cr1GFP, Ccr2RFP) in the desired mouse strain. (c) A customized RFLP using Bsh1236I (recognizing CGCG sites) was designed based on polymorphisms in exon 2 of Sirpa to distinguish between NOD and C57Bl/6 Sirpa alleles.

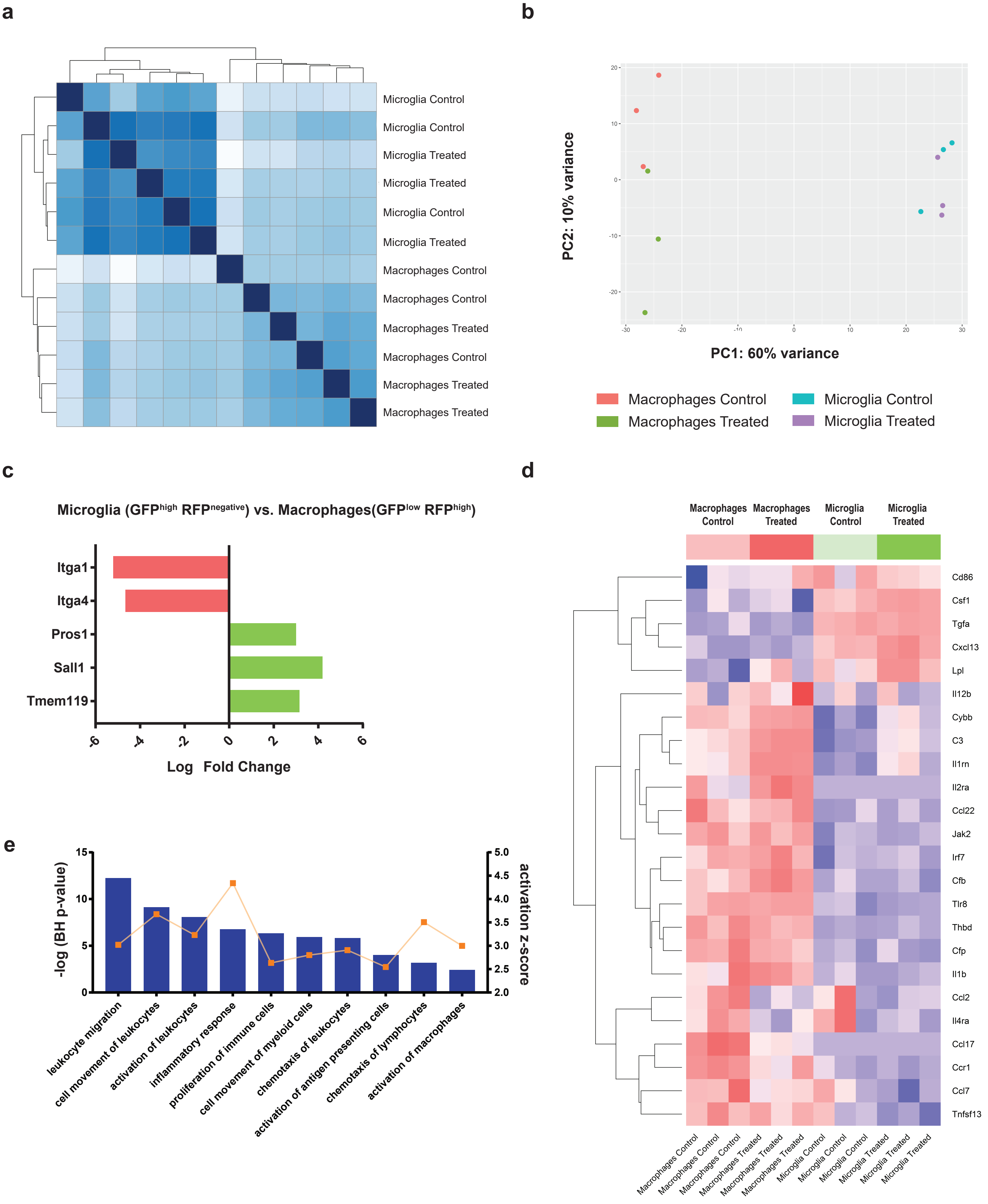

Supplementary Figure 2: Transcriptional Analysis of Tumor-associated macrophages and microglia (a) Heatmap of sample-to-sample distances using the Poisson distance of the rlog-transformed gene expression matrix. (b) Principal-component analysis (PCA) of tumor-associated microglia and macrophages from T387-grafted NSG-Ccr2RFP/wtCx3cr1GFP/wt. Number of highest varying genes used for PCA: 200. (c) Bar graph depicting the log2fold change of FPKM counts of the listed genes of microglia versus macrophages in the control situation (TA-MG n=3, TA-MAC n=3). (d) Heatmap depicting row z-scores of log2 transformed gene expression of TA-MAC control (light red), TA-MAC treated (red), TA-MG control (light green) and TA-MG treated (green). (e) IPA® functional analysis of differentially expressed genes between tumor-associated microglia and macrophages from control treated mice. P-values are Benjamini-Hochberg corrected. Z-score indicates the upregulation of that function in tumor-associated macrophages

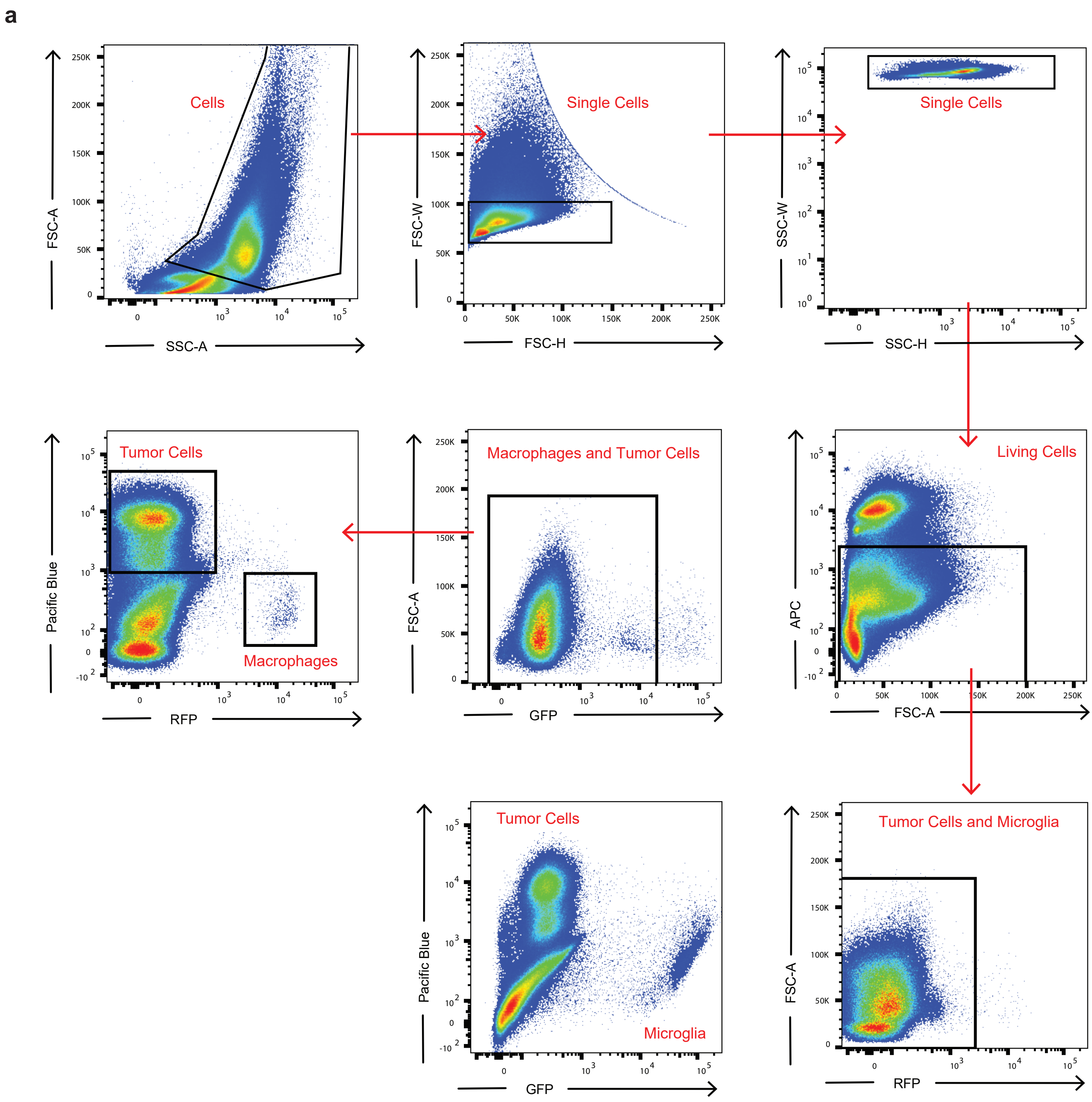

Supplementary Figure 3: FACS gating strategy for Tumor Associated Microglia and Macrophages (a) Debris and doublets were removed. Sytox Red (APC channel) was used to exclude dead cells. For phagocytosis assays, BFPnegative GFPnegative RFPnegative were also excluded. Microglia were defined as GFPhigh RFPnegative whereas macrophages were assessed as GFPlow RFPpositive.

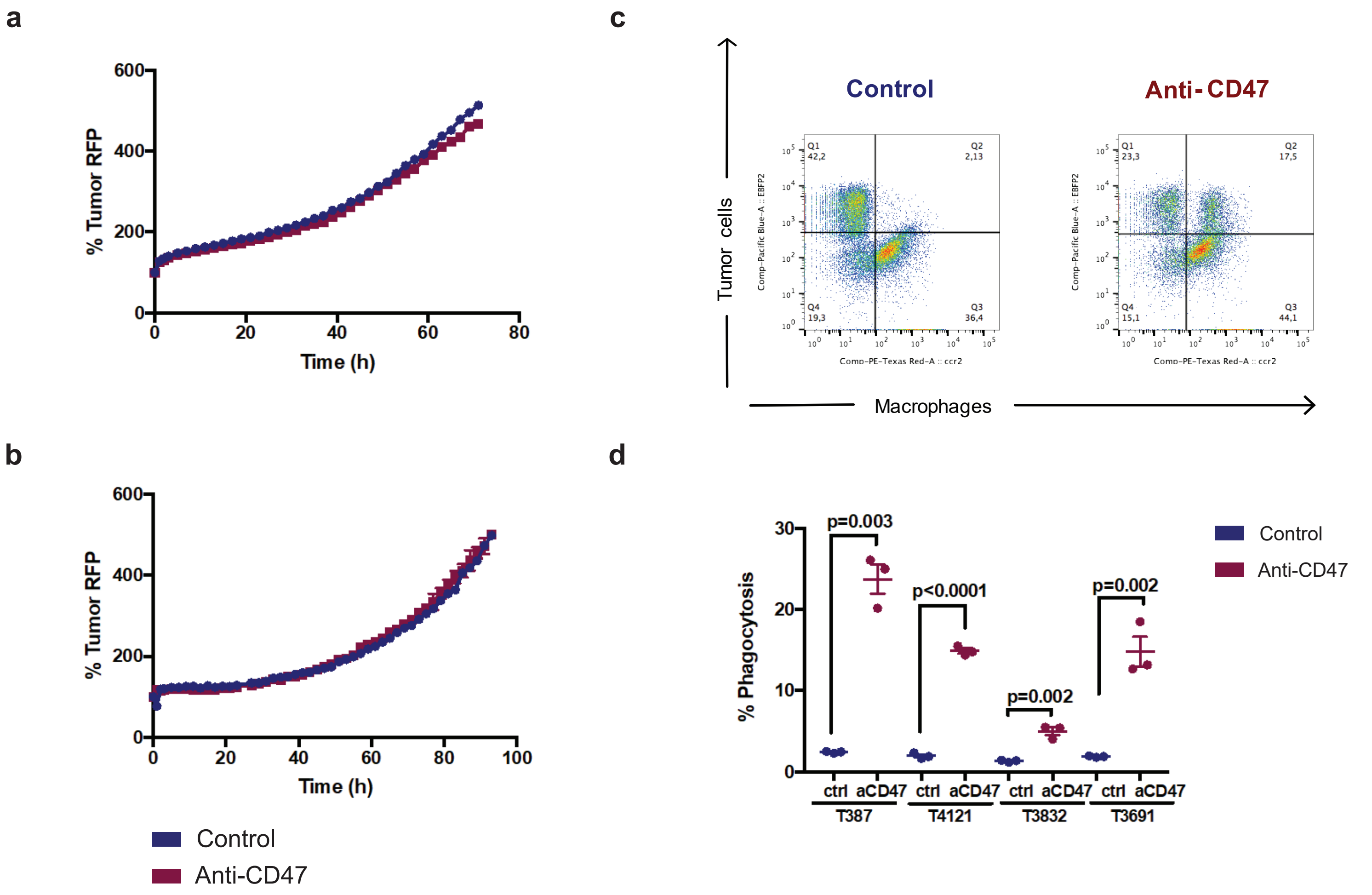

Supplementary Figure 4: Anti-CD47 has no impact on proliferation of the glioma lines T387 and T3832. (a)(b) 10k TdTom+ tumor cells / well were seeded in a 96-well plate and either treated with anti-CD47 (10µg/ml) or control. Red fluorescent signal was obtained every 3 hours. Data was normalized to the first measured value. (c) (d) The same concentration of anti-CD47 led to significant phagocytosis of tumor cells by macrophages in vitro (c) Representative Flow image is shown (d) % of phagocytosing macrophages are shown.

Legend for corresponding videos

Movie S1

In vivo confocal single plane time series of the tumor region of an untreated mouse. Blue – Angiosense680+ blood vessels; green – microglia; red – macrophages.

Movie S2

In vivo confocal single plane time series of the tumor region of an anti-CD47 treated mouse. Blue – Angiosense680+ blood vessels; green – microglia; red – macrophages. Note active macrophage extravasation into the tumor bed.

Movie S3 and Movie S4

Single plane 90 minutes time series (a) and closeup (b) of active microglial tumor cell phagocytosis and macrophage-microglial interaction under anti-CD47 treatment. Close-up shows a microglial cell migrating to the tumor cells and phagocytizing it over the course of the movie.

Movie S5

Single plane 60 minutes time series of tumor-associated microglia and absence of microglial phagocytosis in an untreated mouse (note sparse/absent macrophage infiltrate).

Movie S6

Combined Z-stack (10µm) 90 minutes time series of single tumor cells invading the contralateral hemisphere under continuous microglial scanning, but without microglial tumor cell phagocytosis (note upcoming tumor cell in the middle of the field of view in the second half of the recording).

Movie S7

Filament tracking reconstruction of microglial processes time series in an untreated mouse.

Movie S8

Filament tracking reconstruction of microglial processes time series in an anti-CD47 treated mouse.

Supplementary Tables

Supplementary table 1. Cell lines used in this study

Cell line name	Origin	Passage type	Subtype profile
T387-EBFP2$^+$Luc$^+$	Dr. Jeremy Rich	High passage	Mesenchymal
CT-2A-Luc$^+$	Dr. Thomas Seyfried	High passage	Murine GBM

Supplementary table 2. Primers for mouse genotyping.

***Sirp*α exon 2**			
SirpExon2fw	TGG CTG CCA TCT TTC TT	Sirpα exon 2	402 bp
SirpExon2rev	TGA AGA AGG CAC AGG CTT ACT	Bsh1231 cuts only homozygous NOD Sirpα	
		Two fragments	112 and 290 bp
***Prkdc*^{scid}**			
Scid F	GAGAAAAGGAGGATCATGGATTCAAGAAATAAATGTAACG	Common (F/R)	210 bp (common)
WR	TGGCCCCTGCTAACTTTCTCTTAGCA	*Scid* specific (MF/R)	162 bp
MF	TGGTATCCACAACATAAAATACGCTAA	*Prkdc* specifc (F/WR)	72 bp
Scid R	CCTAAGAGTCACTTTCTCCATTTACACAGTGAAGTGCC		
***Ccr2*^{RFP}**			
13505	TAA ACC TGG TCA CCA CAT GC	wildtype	494 bp
13506	GGA GTA GAG TGG AGG CAG GA	mutant	320 bp
13507	CTT GAT GAC GTC CTC GGA G	heterozygote	320 bp and 494 bp
Cx3cr1			
14276	GTC TTC ACG TTC GGT CTG GT	wildtype	410 bp
14277	CCC AGA CAC TCG TTG TCC TT	mutant	500 bp
14278	CTC CCC CTG AAC CTG AAA C	heterozygote	410 and 500 bp
Il2rg			

19475	AAG AGA TTA CTT CTG GCT GTC AG	wildtype	94 bp
19477	CTC TGG GGT TTC TGG GG	mutant	130 bp
oIMR3914	ATG CTC CAG ACT GCC TTG	heterozygote	94 bp and 130 bp

Supplementary table 3: Antibodies

Antigen	Fluorescent Dye	Supplier
CD45	PE-Cy7	BioLegend, San Diego, USA
CD11b	PerCP-Cy5.5	BioLegend, San Diego, USA
F4/80	Alexa Flour 700	BioLegend, San Diego, USA
Ly6G	Alexa Flour 647	BioLegend, San Diego, USA
Ly6C	APC	BioLegend, San Diego, USA
